# Postprandial effect of dietary fat quantity and quality on arterial stiffness and wave reflection: a randomised controlled trial

**DOI:** 10.1186/1475-2891-12-93

**Published:** 2013-07-10

**Authors:** Fiona E Lithander, Louise K Herlihy, Deirdre M Walsh, Emma Burke, Vivion Crowley, Azra Mahmud

**Affiliations:** 1 Department of Clinical Medicine, Trinity College Dublin, Trinity Centre for Health Sciences, St James’s Hospital, James’s Street, Dublin 8, Ireland; 2 ANU Medical School and The John Curtin School of Medical Research, The Australian National University, Canberra ACT 0200, Australia; 3 Department of Biochemistry, St James’s Hospital, James’s Street, Dublin 8, Ireland; 4 Department of Pharmacology and Therapeutics, Trinity College Dublin, Trinity Centre for Health Sciences, St James’s Hospital, James’s Street, Dublin 8, Ireland

**Keywords:** Arterial stiffness, Wave reflection, Postprandial, Dietary fat, Monounsaturated fat, Saturated fat, Pulse wave velocity, Augmentation index

## Abstract

**Background:**

Arterial stiffness is a component of vascular function and an established risk factor for cardiovascular disease. There is a lack of conclusive evidence on the effect of a meal rich in monounsaturated fat (MUFA) compared with an isoenergetic meal rich in saturated fat (SFA) on postprandial vascular function and specifically on arterial stiffness.

**Methods:**

Twenty healthy, non-smoking males (BMI 24 ± 2 kg/m^2^; age 37.7 ± 14.4 y) participated in this single-blind, randomised, cross-over dietary intervention study. Each subject was randomised to receive a high-fat test-meal (3 MJ; 56 ± 2 g fat) at breakfast on 2 separate occasions, one rich in oleic acid (MUFA-meal) and one rich in palmitic acid (SFA-meal), and the meals were isoenergetic. Blood pressure (BP), arterial stiffness (PWV) and arterial wave reflection (augmentation index, AIx) were measured using applanation tonometry at baseline and every 30 minutes up to 4 hours after the ingestion of the test-meals.

**Results:**

All subjects completed both arms of the dietary intervention. There was no significant difference in BP parameters, PWV or AIx at baseline between the two treatments (*P* > 0.05). There was a significant increase in brachial and aortic BP, mean arterial pressure (MAP), heart rate and PVW (time, *P* < 0.05) over the four hours after consumption of the fat-rich test-meal although the increase in PWV was no longer significant when adjusted for the increase in MAP. There was no difference in PWV between the two treatments (treatment*time, *P* > 0.05). There was a significant reduction in AIx (time, *P* < 0.05) over the four hour postprandial period although this was no longer significant when adjusted for the increase in heart rate and MAP (time, *P* > 0.05). There was no difference in AIx between the two treatments (treatment*time, *P* > 0.05). However, the reduction in heart rate corrected augmentation index (AIx_75_) was significant when corrected for the increase in MAP (time, *P* < 0.01) with no differential effect of the treatments (treatment*time, *P* > 0.05).

**Conclusions:**

This study has demonstrated a BP dependent increase in PWV and a decrease in arterial wave reflection in the four hour period in response to a high-fat meal. There was no evidence however that replacement of some of the SFA with MUFA had a differential effect on these parameters. The study highlights the need for further research to understand the effects of the substitution of SFA with MUFA on non-serum, new and emerging risk factors for CVD such as arterial stiffness.

## Introduction

Dietary fat intake plays a significant role in the development of cardiovascular disease (CVD) through its effect on lipoprotein metabolism [1]. It is well accepted that dietary saturated fatty acid (SFA) is positively associated with cardiovascular (CV) risk and whilst current dietary recommendations advise a decrease in SFA intake [2,3], it is unclear which nutrient should replace SFA in the diet [4]. The chronic substitution of SFA with monounsaturated fat (MUFA) is attractive since a decrease in serum markers of CVD risk such as fasting LDL-cholesterol has been shown in a number of studies [5,6]. It is also becoming increasingly evident that since humans spend the majority of each 24 hour period in the postprandial state, an understanding of the acute metabolic consequences of a meal is important [7,8]. Indeed, it is well accepted that an adverse lipaemic state in the postprandial phase is itself a risk factor for CVD [7].

The examination of the effects of fat quantity and SFA replacement on non-serum markers of CVD is important and vascular dysfunction is a new and emerging non-serum risk factor for CVD. Arterial stiffness, measured as aortic pulse wave velocity (PWV) is a validated, independent predictor of mortality from CVD [9] and is an unavoidable element of CV aging [10]. Whilst a number of studies have examined the postprandial effect of a fat-rich meal on components of vascular function such as flow-mediated dilatation (FMD) and forearm blood flow (FBF) in healthy subjects [11-24], there is conflicting evidence on the effect on arterial stiffness which is dependent on the methodology used [25-27]. Moreover, there is a lack of conclusive evidence on the acute effect of a test-meal rich in monounsaturated fat (MUFA) compared with an isoenergetic test-meal rich saturated fat (SFA) on postprandial vascular function but specifically on arterial stiffness [8]. Given the public health recommendations in favour of dietary SFA replacement [2,3], it is important to understand the impact of such substitution on new and emerging risk factors for CVD [8]. The aim of the current study was twofold; to investigate the acute postprandial effects of a fat-rich meal compared to baseline and secondly to examine the effect of a MUFA-rich versus a SFA-rich meal on postprandial arterial stiffness and wave reflection.

## Methods

### Study population

Twenty men completed both arms of this dietary intervention. All had normal fasting lipid profile, plasma glucose and serum insulin, were normotensive and were not current cigarette smokers (Table 1). Exclusion criteria included a self-reported history of coronary heart disease, any metabolic disorder, diabetes mellitus, atrial fibrillation, heart failure, renal or liver dysfunction, secondary hypertension; currently or previously being treated for hypertension; body mass index > 30 kg/m^2^; resting blood pressure (BP) > 140/90 mmHg; inability to consume the test-meal through dysphagia or intolerance; taking medications or currently participating in another intervention study. Subjects were asked to abstain from taking any nutritional supplements for the duration of the study and for 3 weeks prior to the intervention. The study was ethically approved by the Trinity College Dublin Faculty of Health Sciences Ethics Committee and each subject gave informed written consent. The study was carried out in accordance with the principles of the Declaration of Helsinki and the procedures followed were in accordance with institutional guidelines.

**Table 1:** Baseline characteristics of the twenty subjects who completed the study

**n**	**20**
Age (years)	38.7 ± 14.4
Body mass index (kg/m^2^)	24.1 ± 2.3
Waist circumference (cm)	85.7 ± 7.0
Brachial systolic BP (mm Hg)	112.0 ± 12.7
Brachial diastolic BP (mm Hg)	69.0 ± 9.2
Heart rate (bpm)	56.2 ± 10.2
Aortic systolic BP (mm Hg)	99.4 ± 14.5
Aortic diastolic BP (mm Hg)	67.8 ± 9.3
Augmentation index (%)	8.4 ± 24.2
Augmentation index at HR of 75 bpm (%)	0.1 ± 7.9
Reflection time (m/s)	157.2 ± 24.8
Pulse wave velocity (m/s)	6.7 ± 1.4
Plasma glucose (mmol/L)	4.85 ± 0.37
Insulin (mU/L)	4.26 ± 2.27
Total cholesterol (mmol/L)	4.5 ± 0.8
High density lipoprotein cholesterol (mmol/L)	1.4 ± 0.3
Triglyceride (mmol/L)	1.0 ± 0.7

Abbreviations: *BP* blood pressure, *bpm* beats per minute, *m/s* metres per second, all measurements were made at the screen visit; all blood parameters were measured after an overnight fast.

### Study protocol and diets

Study subjects in this randomised, controlled, single-blind, cross-over design were required to attend the Trinity Centre for Health Sciences on three separate occasions for one screen visit and two test-meal visits. Eligibility was assessed at the screen visit. Subjects were asked to arrive at 0800 h having fasted from 2000 h the previous night and also having abstained from vigorous exercise and consumption of alcohol and caffeine for the previous 24 hours. Subjects were randomised to the two separate test-meals using stratification to ensure that half were given the test-meal which was rich in monounsaturated fatty acids (MUFA-meal) and low in SFA, and half the test-meal which was rich in SFA (SFA-meal) and low in MUFA on entry into the study. Each subject therefore received each of the two test-meals on a separate occasion and in a randomised order. There was a minimum washout period of two days between the two test-meal visits where subjects were asked to resume their habitual diet and exercise patterns.

During each test-meal visit, subjects remained supine apart from short periods in between measurements where they were permitted to sit up, read or visit the bathroom. They were not permitted to fall asleep at any stage. Measurements of BP, PWV and arterial wave reflection measured as augmentation index (AIx) were made at 9 individual time-points; while the subject was fasting (0 minutes) and at 30, 60, 90, 120, 150, 180, 210 and 240 minutes after the start of ingestion of the test-meal.

Both isoenergetic test-meals comprised of a high-fat (3 MJ, 56 ± 2 g fat) strawberry flavoured milkshake and 400 ml water which subjects were asked to consume in entirety within 15 minutes. The MUFA-meal contained whole milk, skimmed milk powder, Nesquik® (strawberry flavour), water and olive oil. The SFA-meal contained whole milk, skimmed milk powder, Nesquik® (strawberry flavour), water, double cream and sunflower oil. The MUFA-meal was rich in oleic acid which came from olive oil. The SFA-meal was rich in palmitic acid, myristic acid and stearic acid which came from the dairy cream. The two milk-shakes were freshly prepared on the morning of each visit and were identical in volume, taste and appearance and in macronutrient composition, other than a difference in the MUFA and SFA content (Table 2).

**Table 2:** Composition of the two test-meals

	**MUFA-meal**	**SFA-meal**	**Delta**
Energy (kJ)	2978.9	3124.6	145.7
CHO (g)	39.7	41.1	1.4
Protein (g)	17.4	18.8	1.4
Fat (g)	54.5	57.6	2.7
**Total SFA (g)**	**11.7**	**33.84**	**22.14**
*SFA profile(g)*			
C4:0	0.28	2.12	1.84
C6:0	0.18	1.22	1.04
C8:0	0.1	0.68	0.58
C10:0	0.22	1.45	1.23
C11:0	0	0.03	0.03
C12:0	0.35	1.73	1.38
C13:0	0	0.03	0.03
C14:0	0.87	5.31	4.44
C15:0	0.08	0.56	0.48
C16:0	6.87	14.29	7.42
C17:0	0.09	0.36	0.27
C18.0	2.23	5.9	3.67
C20:0	0.19	0.09	0.1
C22:0	0.05	0.06	0.01
C24:0	0.19	0.01	0.18
**Total MUFA (g)**	**36.42**	**14.64**	**21.78**
*MUFA profile(g)*			
C10:1	0.02	0.14	0.12
C12:1	0	0.03	0.03
C14:1	0.08	0.45	0.37
C16:1	0.47	0.83	0.36
C17:1	0.07	0.12	0.05
C18:1	35.4	11.43	23.97
C20:1	0.16	0.13	0.03
C22:1	0	0	0
C24:1	0	0.03	0.03
Trans MUFA (g)	0.22	1.48	1.26
**Total PUFA (g)**	**3.97**	**4.18**	**0.21**
*PUFA profile(g)*			
C18:2	3.59	3.39	0.2
C18:3	0.34	0.27	0.07
C20:2	0	0.01	0.01
C20:3	0	0.03	0.03
C20:4	0	0.06	0.06
C20:5	0	0.03	0.03
C22:5	0	0.04	0.04
Trans PUFA (g)	0.04	0.35	0.31

Abbreviations: *CHO* carbohydrate, *SFA* saturated fatty acid, *MUFA* monounsaturated fatty acid, *PUFA* polyunsaturated fatty acid.

### Haemodynamic measurements

BP and heart rate were measured in duplicate prior to each set of arterial stiffness measurements in the right arm of each subject using an A&D Medical UA-767 Plus digital blood pressure monitor (Tokyo, Japan) using a medium sized cuff. All observers were trained by the senior investigator AM. A pillow was placed under the subjects’ right arm in order to raise the brachial artery to the level of the aorta. The measurement was repeated if there was a difference of ≥ 10 mmHg between two values and the two closest values were recorded.

Applanation tonometry was performed using SphygmoCor® (Model SCOR-Px, AtCor Medical, Sydney, Australia) on the right side of each subject to derive values for PWV and AIx at the screen visit and at the 9 timepoints at each of the test-meal visits. An ECG was performed for the duration of each testing period. At the screen visit the distance from the suprasternal notch to the femoral artery and the distance from the suprasternal notch to the carotid artery were measured using a flexible tape measure and these data used for the test-meal visits. The length of the aorta was estimated as the distance from the suprasternal notch to the femoral artery minus the distance from the suprasternal notch to the carotid artery. Applanation tonometry commenced two minutes after the deflation of the blood pressure monitor cuff. A measurement of arterial stiffness was taken using the tonometer at the carotid and femoral arteries which gives carotid-femoral PWV. Pulse wave analysis (PWA) was performed at the radial artery of the right arm of each subject. AIx, aortic systolic blood pressure (ASBP) and aortic diastolic blood pressure (ADBP) were derived from PWA. Furthermore, as AIx is intrinsically heart rate dependent, the system also generated AIx_75_ which standardises AIx to a heart rate of 75 beats per minute.

### Anthropometry

The body mass of each subject was measured in kilograms to the nearest 0.1 kg on SECA 877 digital scales (SECA, Vogel and Halke, Germany) with the subject wearing light clothing and no shoes. Height was measured to the nearest millimetre using a SECA Leicester portable stadiometer (SECA, Vogel and Halke, Germany) with the subject standing upright, with their head in the Frankfort plane and the shoulders and buttocks in contact with the backboard. Waist circumference was measured to the nearest millimetre using a flexible tape measure at the midpoint between the lowest rib and the iliac crest after respiratory exhalation and the subject standing with feet approximately 20 cm apart.

### Statistical analysis

Results are expressed as mean ± SD unless otherwise stated. Data were normalised and were analysed using JMP Version 7.1.1 (SAS for Windows, NC, USA). The sample size was calculated on the assumption that with a within-patient standard deviation of PWV of 0.9, 20 subjects would be required in a cross-over study with a probability of 91% to detect a treatment difference of 1 m/sec at a two-sided 0.05 significance level. Continuous data is expressed as mean ± SD and categorical data as percentages. Differences between means were analysed using Wilcoxon Rank Sums Test for continuous and chi-square test for categorical data. Changes in clinical parameters over time were analysed using ANOVA for repeated measures with treatment as a covariate. For PWV, which is BP dependent, changes over time were also adjusted for the change in MAP. For AIx, changes over time were adjusted for a change in heart rate and in MAP. A significance level of *P* < 0.05 was considered significant.

## Results

All subjects who were randomised into this cross-over study completed both arms of the intervention. Subjects were healthy and predominantly lean (BMI 24.1 ± 2.3 kg/m^2^). Baseline characteristics are reported in Table 1. There was no significant difference in PWV, AIx, brachial systolic blood pressure (SBP) or aortic SBP at the fasting measure (0 minutes) between the two treatments (*P* > 0.05). All subjects had normal fasting glucose (4.85 ± 0.37 mmol/L) and insulin (4.26 ± 2.27 mU/L), were normotensive with no evidence of hyperlipidaemia.

### Effect of the test-meal fat quantity on PWV, AIx and BP

When analysed independent of treatment, there was a significant increase in brachial (time, *P* < 0.05) and aortic SBP (time, *P* < 0.05), MAP (time, *P* < 0.05) and heart rate (time, *P* < 0.001) in response to the high-fat meal over the 240 minute postprandial period. There was also a significant increase in PWV over time (time, *P* < 0.05), although this effect was no longer significant when adjusted for the increase in MAP (*P* > 0.05). AIx decreased significantly in response to the high-fat meal (time, *P* < 0.01), although this effect was no longer significant when adjusted for the increase in heart rate and MAP (*P* > 0.05). There was a significant reduction in AIx_75_ over the four hour postprandial period (time, *P* < 0.01) which was still significant when corrected for the increase in MAP (*P* < 0.05).

### Effect of the test-meal fatty acid quality on PWV, AIx and BP

**Figure 1:**
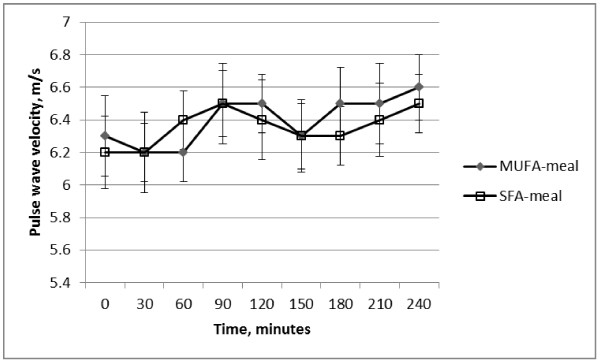
Change in pulse wave velocity during the 240 minute intervention period, mean ± sem, n = 20.

**Figure 2:**
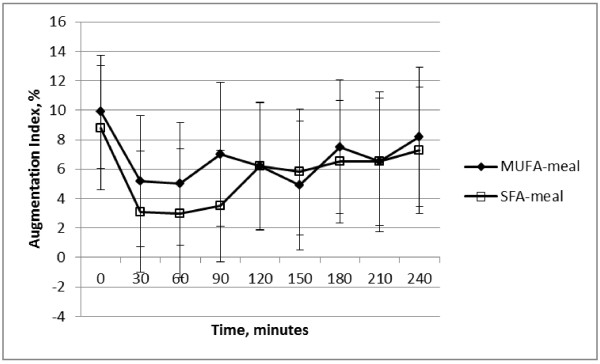
Change in augmentation index during the 240 minute intervention period, mean ± sem, n = 20.

The changes in PWV and in AIx in response to the two test-meal treatments are shown in Figure 1 and Figure 2. When analysed to investigate the effect of the MUFA-meal versus the SFA-meal, there was no significant differential effect of the MUFA-meal versus the SFA-meal on brachial or aortic SBP, MAP or heart rate (treatment*time, *P* > 0.05). There were also no differential effects of fatty acid quality on PWV, AIx, or AIx_75_ (treatment*time, *P* > 0.05).

## Discussion

It is widely accepted that there is a positive relationship between the fat content of a meal and the resultant lipaemia [28], inflammation [29], coagulation and oxidative stress [30,31]. The current study demonstrated a significant increase in arterial stiffness measured as PWV with a significant decrease in wave reflection measured as AIx compared to baseline in the four hour period in response to a high fat meal. No differential effect was seen on either PWV or AIx when some of the SFA in the SFA-meal was substituted for MUFA.

### Effect of the test-meal fat quantity on postprandial arterial stiffness and wave reflection

A significant increase from baseline of 0.3 m/s in PWV following the high-fat meal was seen in the current study although this was not significant when the data were adjusted for the increase in MAP. The meal provided 56 g dietary fat and data from a number of other studies of lean healthy men [32,33] demonstrate an increase in serum triacylglycerol rich lipoproteins (TRLs) in response to a similar amount of dietary fat. It has been suggested that this hypertriglyceridaemic state may affect non-serum risk factors for CVD such as vascular function [34]. A number of studies have examined the acute effect of fat quantity on postprandial vascular function in healthy participants [12-24,35,36] and although there were methodological differences between the studies, results concluded an impairment in vascular function following a meal rich in fat [8]. The majority of these studies used flow mediated dilation (FMD) or forearm blood flow (FBF) as their measure of vascular function. Only three studies have examined the acute effect of fat ingestion where arterial stiffness was the primary outcome measure but suffer from methodological differences [25-27]. The measurement of carotid-femoral PWV that was used in the current study is widely recognised as the gold-standard method of assessing arterial stiffness. The first of these three studies demonstrated an impairment in systemic arterial compliance in response to 50 g fat [27]. The second showed no change in PWV, pulse wave analysis (PWA) or digital volume pulse (DVP) in response to 60 g fat [25] although carotid-radial PWV was used which has not been shown to be of prognostic value and is not recommended for assessing arterial stiffness. The third study by Phillips and colleagues (2010) showed an acute decrease in AIx after 58 g fat was consumed [26]. Two of the studies were carried out in lean and obese men [25,26] with no differential effect of body mass index (BMI). The third study [27] was carried out in both men and women although BMI was not reported. While one may argue that the increase in PWV shown in the current study was BP dependent and therefore not significant, we believe that this unfavourable acute haemodynamic effect of a high-fat meal does support a view of a pro-atherogenic postprandial milieu.

The mechanism proposed by Nestel and colleagues [27] is that postprandial serum TRLs contribute to arterial stiffening, a hypothesis that is put forward by the authors of the current study. Others have observed this association and have postulated that an increase in lipolysis may increase the production of reactive oxygen species (ROS) and increase the possibility that circulating fatty acids may become oxidised [37]. In addition, the ROS can decrease the bioavailability of nitric oxide (NO) [18,20], either through decreased synthesis and/or enhanced degradation [38]. Through effects on coagulation and inflammatory signalling pathways in the endothelium, circulating TRL remnants which result from a high-fat meal may increase the breakdown of NO [39,40]. Serum TRLs were not measured in the current study although it is hypothesised that significant hypertriglyceridaemia occurred in response to the 56 g fat consumed which had either a direct or indirect effect on vascular health.

Most published work shows a reduction in wave reflection following a high-fat meal. In the current study, there was a significant decrease in AIx compared to baseline in response to the high-fat meal, although this disappeared when adjusted for the increase in heart rate and MAP. However, AIx_75_, which is corrected for heart rate, significantly decreased following the high-fat meal, independent of a change in MAP. This reduction is counter intuitive when we consider data showing impaired endothelial function following the acute ingestion of dietary fat and larger studies are required to improve our understanding. One of the hypotheses put forward to explain this paradox includes the proposal that the ingestion of any type of food causing a reduction in wave reflection from the splanchnic bed [41] which may be related to the release of insulin, a known vasodilator [42].

### Effect of the test-meal fatty acid quality on postprandial arterial stiffness and wave reflection

The second aim of the current study was to assess whether replacement of dietary SFA with MUFA would affect postprandial arterial stiffness measured as PWV and wave reflection measured as AIx, and results indicated that no differential effect was seen over the 4 hour intervention period. When serum markers of CVD risk are measured, it is well accepted that an MUFA-rich diet results in a less atherogenic profile compared to a SFA-rich diet [43]. A number of studies have investigated the effect of isoenergetic meals of different fatty acid composition on measures of vascular function and substantial differences in study design has led to difficulties in drawing conclusions [8]. In addition, some studies tested healthy subjects [38,44-48] whilst other investigated hyercholesterolaemic patients [49], type two diabetics [50,51] or adults with the metabolic syndrome [52]. The overall evidence suggests a moderate decrease in vascular function following a SFA-rich meal [8] but the data are less consistent when SFA is replaced by MUFA. Importantly, the majority of studies used FMD, FBF or ischaemic reactive hyperaemia [52] as measures of vascular function and only one study [38] examined the effect on arterial stiffness measured as PVW and/or PWA. Berry and colleagues [38] tested the postprandial effect of a stearic acid rich meal (50 g fat) versus an oleic-acid rich meal (50 g fat) and showed a decrease in PWV and PWA after both meals when compared with baseline. Such results should be interpreted with caution however because although stearic acid is correctly classified as a SFA, it can result in an uncharacteristically low postprandial lipaemic and oxidative stress response [38] when compared to a meal rich in the MUFA oleic acid. In the current study, the main sources of SFA in the SFA-meal were palmitic acid and myristic acid and therefore this issue was avoided.

The current study showed a significant increase in both brachial and aortic SBP in response to the high-fat meal but no differential effect of fatty acid quality on either measure. It is unknown however whether the increased systolic blood pressure was caused by the increase in PWV or vice versa. Increased blood pressure in response to a high-fat meal has been seen elsewhere [53] and it is hypothesised that the fat-load-induced increase in circulating free fatty acids and TRLs stimulate production of ROS [53,54], and contribute to endothelial dysfunction [55], hypothesised to contribute to hypertension. In the current study, whether the meal was rich in MUFA or SFA had no differential effect on either brachial or aortic systolic blood pressure.

It could be argued that the changes in vascular function observed over the 4 hour postprandial period in the current study may be as a result of eating *per se* rather than dietary fat intake. Studies have shown that dietary intake, regardless of the composition, can cause a change in arterial stiffness [56-58]. Eating is known to cause changes in a number of hemodynamic responses [59] although these tend to lead to vasodilation rather than vasoconstriction [27], as previously discussed. Studies where carbohydrate has replaced fat have resulted in improved endothelial function [13]. Whilst the current study allowed us to test the postprandial effect of a fat rich meal compared to baseline, it was not specifically designed with this in mind and as such did not use a control isoenergetic low-fat/high carbohydrate meal. The other components of any test-meal can make the interpretation of the resultant data somewhat complex. The protein [60], anti-oxidant [61,62] and fibre content [63] of test-meals can complicate the interpretation of data and make it difficult for conclusions to be drawn on the effect of fats of differing quality. The test-meals used in the current study were identical in volume, taste, appearance and in macronutrient composition other than a difference in MUFA and SFA content. One limitation in the current study however, is that the MUFA-meal contained olive oil which is known to contain phenolic compounds. Ruano and colleagues (2005) showed that a meal containing high-phenolic virgin olive oil improves ischemic reactive hyperaemia during the postprandial state, a phenomenon which might be mediated via a reduction in oxidative stress and the increase of nitric oxide metabolites [64]. The phenolic content of the olive oil used in the current study was not measured and therefore could have been a confounding factor. In addition, it would have been preferable if the intervention period was longer than 240 minutes since studies and a recently published expert panel statement suggest that many serum markers take greater than 240 minutes to return to baseline after a high-fat meal [26,65].

Another limitation of the current study is that chronic or habitual dietary intake was not measured. In a review published by Lopez-Miranda and colleagues in 2007 [66], it was suggested that the habitual diet of an individual may influence the postprandial response [67] Williams [67] suggested that background diets rich in monounsaturated fat or omega-3 polyunsaturated fatty acids tend to lower the postprandial lipid response compared with diets rich in saturated fatty acids [68-70]. The key limitation of the current study however is the lack of simultaneous collection of venous blood samples. This would have allowed the measurement of circulating TRLs, insulin, markers of oxidative stress and other parameters which would facilitate a greater understanding of the mechanism behind the relationship between dietary fat and arterial stiffness.

## Conclusion

In conclusion, given the known health risks associated with excess dietary fat intake in addition to the health benefits of the replacement of SFA with MUFA on serum markers of CVD risk, further research is needed to understand the effect that dietary fat quantity and quality may have on vascular health.

## Competing interests

The authors declare that they have no competing interests.

## Authors’ contributions

FEL was the senior author, conceived and designed the research, drafted the manuscript and handled funding and supervision; LKH, DMW and EB were responsible for participant recruitment and acquired, analysed and interpreted the data; VC was the senior laboratory analyst and participated in study design; AM was the study clinician, performed the statistical analyses and made critical revision of the manuscript for intellectual content. All authors read and approved the final manuscript.
